# Dietary guidance normalizes large intestinal endocrine cell densities in patients with irritable bowel syndrome

**DOI:** 10.1038/ejcn.2015.191

**Published:** 2015-11-25

**Authors:** T Mazzawi, T Hausken, D Gundersen, M El-Salhy

**Affiliations:** 1 Division of Gastroenterology, Department of Medicine, Stord Hospital, Stord, Norway; 2 Division of Gastroenterology, Department of Clinical Medicine, University of Bergen, Bergen, Norway; 3 National Centre for Functional Gastrointestinal Disorders, Department of Medicine, Haukeland University Hospital, Bergen, Norway; 4 Department of Research, Helse-Fonna, Haugesund, Norway

## Abstract

**Background/Objectives:**

To determine the large intestinal endocrine cell types affected following dietary guidance in patients with irritable bowel syndrome (IBS).

**Subjects/Methods:**

The study included 13 IBS patients and 13 control subjects. The patients received three sessions of individualized dietary guidance. Both the control subjects and the patients were scheduled for colonoscopies at baseline and again for the patients at 3–9 months after dietary guidance. Biopsy samples were taken from the colon and rectum and were immunostained for all types of large intestinal endocrine cells. The endocrine cells were quantified using computerized image analysis.

**Results:**

The daily total consumption (mean±s.e.m. values) of fruits and vegetables rich in FODMAPs (fermentable oligosaccharides, disaccharides, monosaccharides and polyols) decreased significantly from 16.2±5.3 g before receiving dietary guidance to 9.2±3.2 g after receiving dietary guidance (*P*=0.02). In the total colon, the densities of serotonin cells were 46.8±8.9, 10.5±2.1 and 22.6±3.2 cells/mm^2^ in control subjects and in IBS patients before and after receiving dietary guidance, respectively (*P*=0.007); the corresponding densities of peptide YY cells were 11.6±1.8, 10.8±1.7 and 16.8±2.1 cells/mm^2^, respectively (*P*=0.06). The cell densities for both serotonin and peptide YY did not change significantly in the rectum. The densities of somatostatin cells in the rectum were 13.5±3.0, 13.2±3.0, and 22.3±3.2 cells/mm^2^ for control subjects and for IBS patients before and after receiving dietary guidance, respectively (*P*=0.01).

**Conclusions:**

The densities of the large intestinal endocrine cells tend to normalize following dietary guidance that may have contributed to the improvement of the patients with IBS symptoms.

## Introduction

Irritable bowel syndrome (IBS) is a common chronic functional gastrointestinal (GI) disorder.^1^ Besides its associated morbidity, IBS patients exhibit a high consumption of health-care resources that represents an economic burden to society as a whole.^1, 2, 3, 4^ There is increasing evidence that IBS can result from dysfunction of the GI neuroendocrine system and GI endocrine cells in particular.^5, 6, 7, 8, 9, 10, 11, 12, 13, 14, 15, 16^

The GI endocrine cells are scattered among the epithelial cells lining the GI lumen.^17, 18, 19^ These endocrine cells interact with the GI luminal contents (especially nutrients) and respond by releasing specific hormones that affect different functions of the GI tract.^5, 20, 21, 22, 23, 24, 25, 26, 27, 28, 29, 30, 31^

Approximately two-thirds of IBS patients relate their symptom development to diet.^32, 33^ Dietary guidance has been proven to reduce the symptoms and improve the quality of life of IBS patients.^34, 35^

A previous study found that changing the dietary intake by the provision of individualized dietary guidance altered the large intestinal total endocrine cells as detected by a general marker for endocrine cells (chromogranin A).^36^ The observed change in chromogranin A cell density after receiving dietary guidance was toward the normal values of controls. However, which endocrine cell type was affected is not clear.^36^

The present study was undertaken to determine the types of large intestinal endocrine cells that are affected after receiving dietary guidance. The study was performed on the same cohort of IBS patients in whom the total population of endocrine cells was observed after receiving dietary guidance.^36^

## Materials and methods

### Patients and controls

Patients of both sexes aged between 18 and 70 years and referred to the Division of Gastroenterology, Stord Hospital, Norway, who fulfilled Rome-III criteria for IBS diagnosis were included in the study. The exclusion criteria were being pregnant or lactating women patients with serious psychiatric or any organic/systemic diseases, drug abuse or previous abdominal surgery, with the exception of appendectomy, caesarean section or hysterectomy. Seven patients did not use any kind of medication. The remaining patients, however, used one or a combination of the following: proton pump inhibitors (*n*=4), thyroxin substitution tablets (*n*=2), asthma inhalators (*n*=1), angiotensin II receptor antagonist antihypertensive tablets (*n*=1), antiallergic tablets (*n*=3), contraceptive pills (*n*=2) and antidepressants/anxiolytics (*n*=2). These patients were instructed not to take any kind of proton pump inhibitors 1 week before starting or during the study.

A control group of 13 subjects was included in the study. They comprised 9 females and 4 males with a mean age of 54 years (range 26–70 years). Four of the control subjects underwent colonoscopies because of GI bleeding that identified the source as hemorrhoids (*n*=3) or angiodysplasia (*n*=1), whereas the other 9 control subjects underwent colonoscopies because of health worries caused by a family member being diagnosed with GI cancer.

The study was performed in accordance with the Declaration of Helsinki and was approved by the local Committee for Medical Research Ethics West, Bergen, Norway. All patients provided both oral and written consents to participate.

### Study design

Forty-six patients (35 females and 11 males) with mean age of 35 years (range 18–69 years) were included in the study. All patients underwent physical examinations, and blood tests were performed to exclude inflammation, infection and other organic diseases. The patients were scheduled for three 45-min sessions of individualized dietary guidance, with a nurse experienced in diet and IBS, at intervals of at least 2 weeks. The patients were examined with colonoscopies before the first session and at 3–9 months (median, 4 months) following the third session of dietary guidance.

### Individualized dietary guidance

Dietary guidance was delivered orally with the help of charts, and in written illustrations. During the first session, the patients received general information about IBS, with emphasis on the importance of a regular and healthy eating pattern and on the food items that worsen IBS symptoms such as insoluble dietary fiber and poorly absorbable FODMAPs (fermentable oligosaccharides, disaccharides, monosaccharides and polyols). The patients were informed that lactose-free milk and other lactose-free dairy products do not provoke IBS symptoms and hence they were allowed to consume such products daily during the study. The patients were supposed to test diets that were either rich or poor in protein, fat or carbohydrate, and to register for 2 weeks in a diary their daily consumption of food and fluids along with any associated symptoms, including the frequency and degree of abdominal pain and abdominal distension and the stool frequency and consistency. The consumption of food supplements containing probiotics, antibiotics and other medications was prohibited during the study unless otherwise specified.

In the second session, the nurse focused mainly on briefly repeating the information given during the first session and on using the patient's diary to identify the food items that triggered IBS symptoms. Based on the obtained information, the patients were asked to alter their diet proportions of protein, fat and carbohydrate, avoid FODMAPs-rich items as well as insoluble fiber and to consume vegetables and fruits that contained lower amounts of FODMAPs and insoluble fiber.

During the third session, each patient gave feedback about the dietary guidance to the nurse, and together with the nurse designed a suitable diet for the patient to follow until the end of the study.

### Dietary assessment

Dietary intake was assessed using the MoBa food frequency questionnaire. This questionnaire reports the frequency and the portion sizes of food stuffs and beverages consumed over a defined period of time. Data analysis was conducted using software to calculate the nutrient content of the diet. The MoBa food frequency questionnaire was developed and validated by the Norwegian Institute of Public Health in Oslo, Norway.^37, 38^ This questionnaire inquires about the consumption of 225 foodstuffs and identifies the dietary habits of the participant, including the intake of any oral supplements, according to typical Norwegian meal patterns. The participants completed the MoBa food frequency questionnaire form before the first session and again at least 3 months after the third session of individualized dietary guidance.^34^

### Colonoscopies, biopsy sampling, histopathology and immunohistochemistry

Both the patients and controls were scheduled to receive colonoscopies, during which four biopsy samples were taken from each segment of the large intestine. The biopsy samples were then divided according to the segment they were taken from into right colon, left colon and rectum, where the right colon referred to the cecum, the ascending colon and the right half of the transverse colon. The left colon referred to the left half of the transverse colon, the descending colon and the sigmoid colon. In the rectum, biopsy samples were taken from the dorsal wall ∼15 cm from the anus.

The biopsy samples were fixed in 4% buffered paraformaldehyde overnight, embedded in paraffin and cut into sections with a thickness of 5 μm. The biopsy samples were examined histopathologically. They were stained with hematoxylin–eosin and immunostained using the Avidin-Biotin complex (ABC) method with the Vectastain ABC kit (Vector Laboratories, Burlingame, CA, USA) and the chromogen 3,3′-diaminobenzidine peroxidase substrate (DAB) kit (Vector Laboratories). The immunostaining method has been described in detail previously.^13^ In brief, the sections were incubated for 2 h with the primary antibodies and then for another 30 min with biotinylated swine anti-mouse IgG (in the case of monoclonal antibodies) or goat anti-rabbit IgG (in the case of polyclonal antibodies), both diluted to 1:200, and for 30 min with ABC diluted to 1:200. The slides were submerged in DAB and counterstained with hematoxylin. Incubation was carried out at room temperature. The primary antibodies used were monoclonal mouse anti-serotonin (code no. 5HT-209, Dako, Glostrup, Denmark), polyclonal anti-porcine peptide YY (PYY; code PYY 11A, Alpha Diagnostic, San Antonio, TX, USA), polyclonal rabbit antisynthetic human pancreatic polypeptide (code no. 114, Diagnostic Biosystems, Pleasanton, CA, USA), polyclonal rabbit anti-porcine oxyntomodulin (code BP508, Acris Antibodies, Herford, Germany) and polyclonal rabbit antisynthetic human somatostatin (code no. A566, Dako); these antibodies were used in dilutions of 1:1500, 1:1000, 1:800, 1:400 and 1:200, respectively.

### Computerized image analysis

The densities of various types of endocrine cell were quantified using computer software Cell^D (Olympus, Tokyo, Japan). The number of immunoreactive endocrine cells and the area of the epithelial cells were measured in 10 randomly chosen fields using a × 40 objective. Each field represented a tissue area of 0.14 mm^2^. The density of endocrine cells is expressed herein as the number of cells per square mm of epithelium. All quantifications were performed by one person (TM) who was not aware of the identity of the slides.

### Statistical analysis

The Kruskal–Wallis nonparametric test with Dunn's test as a post test was used to compare between the controls and patients before dietary guidance and also between controls and patients after dietary guidance. The paired *t-*test was used to compare patients before and after receiving dietary guidance. The data are presented as mean±s.e.m. values. *P*-values of <0.05 were considered significant.

## Results

### Patients and controls

Forty-six patients were included in the study and received sessions of individualized dietary guidance. After the first session and colonoscopy, four patients were excluded after being diagnosed with celiac disease (*n*=2) and lupus (*n*=1) and because of technically difficult colonoscopy (*n*=1). The remaining patients (*n*=42) went through the second session of individualized dietary guidance. Two patients were also excluded during the study because of pregnancy (*n*=1) and moving abroad (*n*=1). Forty patients received the third session of individualized dietary guidance. Following the third session, 24 patients withdrew their consent, either because of symptom improvement after receiving dietary guidance and/or because they were unwilling for a second colonoscopy to be performed. Additional two patients were excluded because of noncompliance (*n*=2). One patient was excluded because of a bout of gastroenteritis under the study (*n*=1). Thus, 13 of the original 46 patients completed the entire study, comprising 8 females and 5 males with a mean age of 34 years (range 20–45 years). The demographic characteristics are summarized in Table 1.

### Dietary assessment

The change in diet in the present study has been described in detail elsewhere.^34^ In brief, the daily total consumption of fruits and vegetables rich in FODMAPs decreased significantly from 16.2±5.3 g before receiving dietary guidance to 9.2±3.2 g after receiving dietary guidance (*P*=0.02). However, the daily consumption of fiber did not differ significantly between before receiving dietary guidance (27.4±2.5 g) and after receiving dietary guidance (23.1±2.2 g, *P*=0.09).^34^

### Colonoscopies, histopathology and immunohistochemistry

The colonoscopies indicated that the colon and rectum were normal both macroscopically and microscopically in patients as well as controls, with no signs of microscopic colitis. Immunoreactive endocrine cells were found in the mucosa of both the colon and rectum of the patients and controls. These cells were either basket or flask shaped, and sometimes exhibited a long basal cytoplasmic process. The numbers of pancreatic polypeptide- and oxyntomodulin (enteroglucagon)-immunoreactive cells in the biopsy samples of the colon and rectum used in the study were too low to allow reliable quantification. There were also too few somatostatin cells in the colon to allow reliable quantification.

### Computerized image analyses

#### Colon

**Figure 1:**
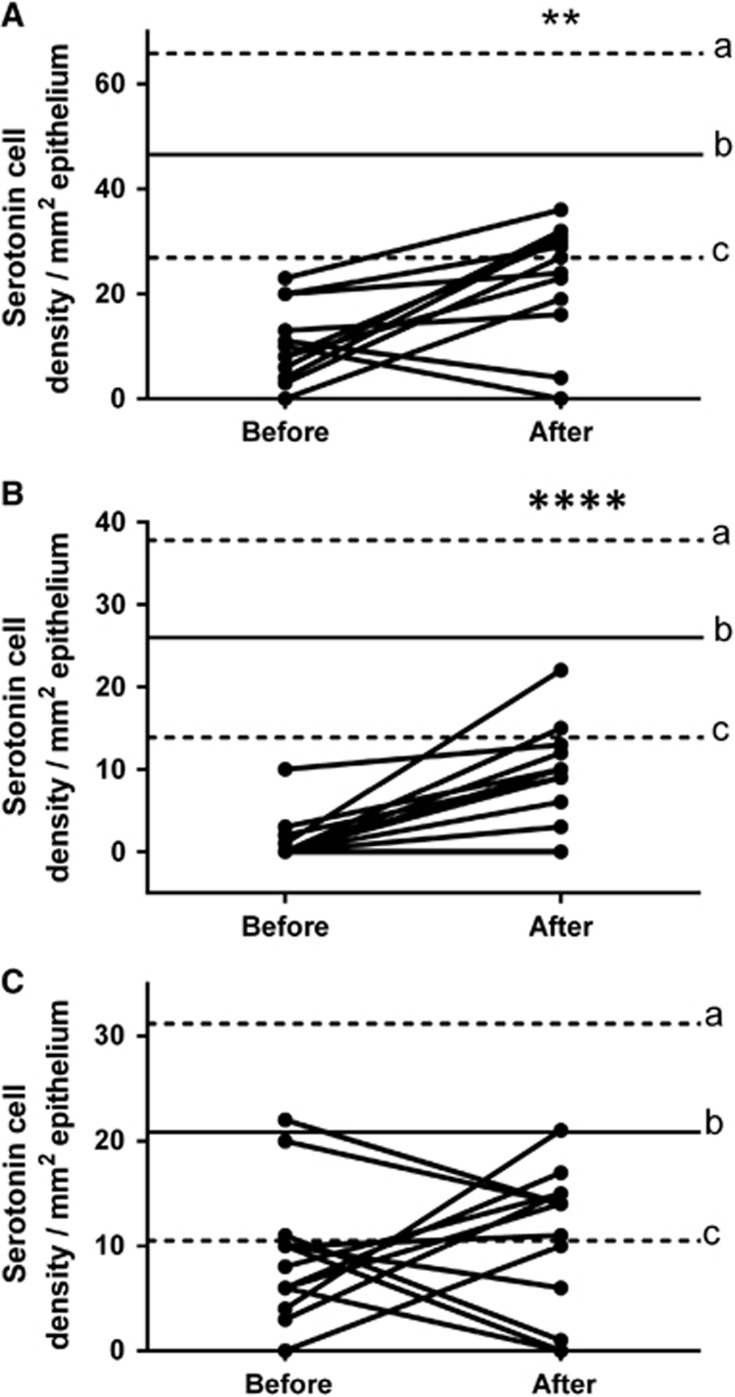
Serotonin cell densities in the total colon (**A**), right colon (**B**) and left colon (**C**) of IBS patients before and after receiving dietary guidance. The dashed lines labeled ‘a' and ‘c' indicate the upper and lower limits of the 95% confidence interval for control subjects, respectively, whereas line ‘b' indicates the mean serotonin cell density. **P<0.01, ****P<0.0001.

**Figure 2:**
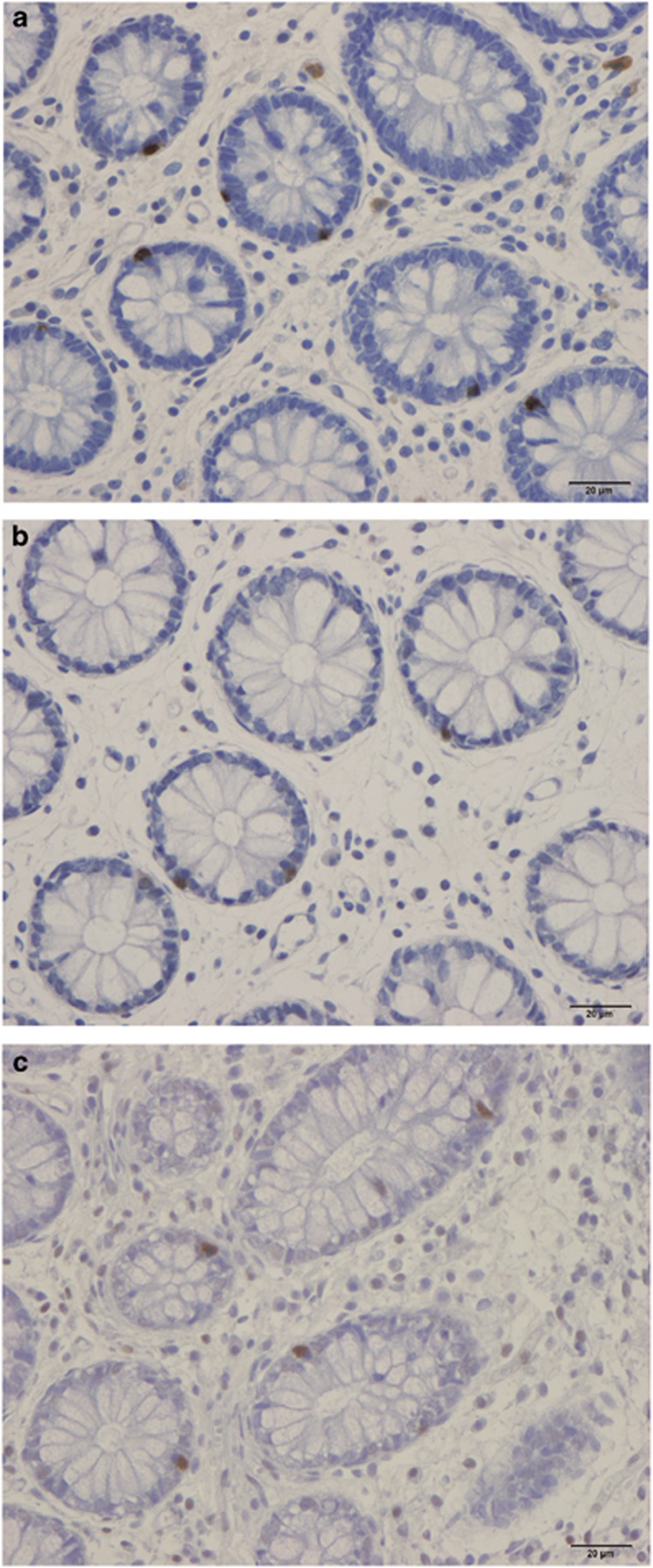
Serotonin-immunoreactive cells in the total colon of a control subject (**a**) and of an IBS patient before (**b**) and after (**c**) receiving dietary guidance.

Serotonin cell density: The densities of serotonin cells in the controls and patients are listed in Table 2 and illustrated in Figures 1 and 2. The density of serotonin cells in the total colon and the right colon of IBS patients increased significantly (*P*=0.007 and *P*<0.0001, respectively) after receiving dietary guidance. The density of serotonin cells in the left colon also increased after receiving dietary guidance, but this increase was not statistically significant (*P*=0.53). The densities of serotonin cells in controls and IBS subtypes before and after receiving guidance are listed in Table 3.

**Figure 3:**
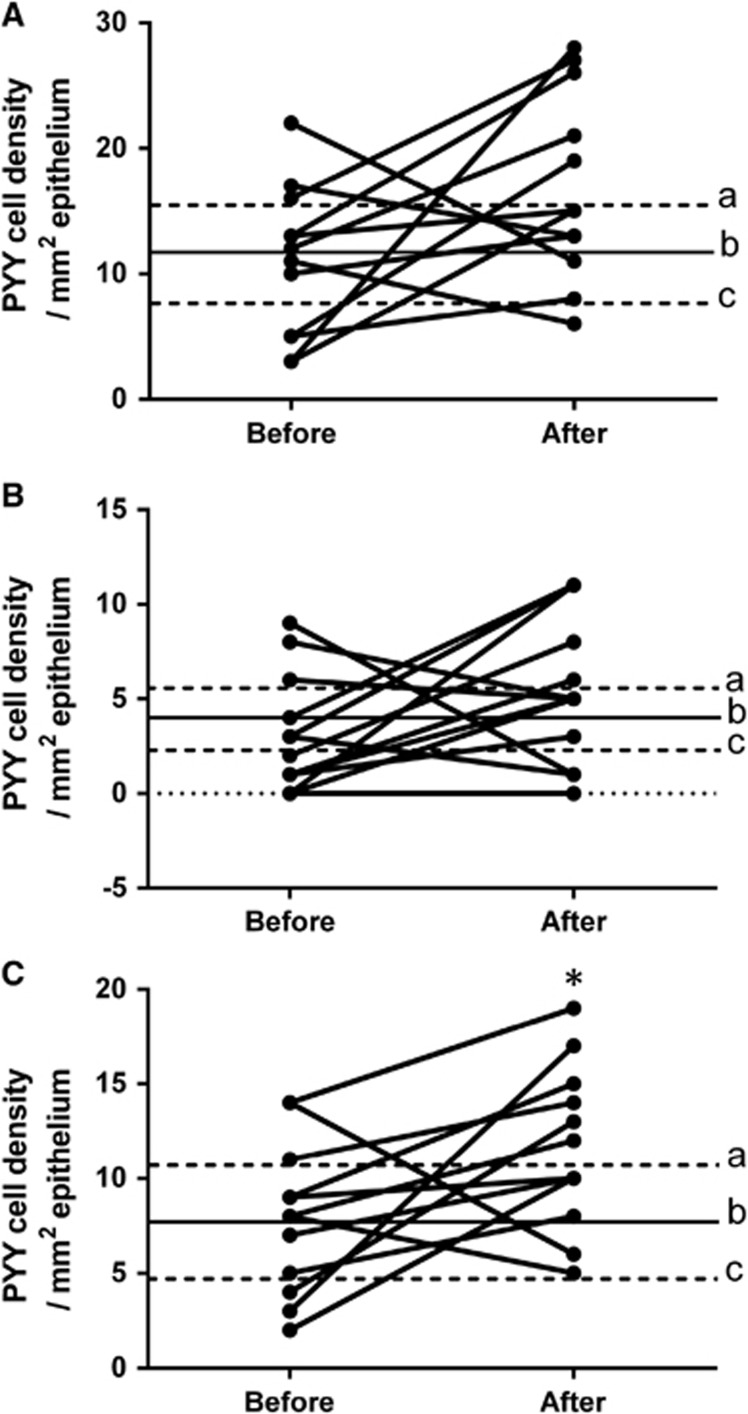
PYY cell densities in the total colon (**A**), right colon (**B**) and left colon (**C**) of IBS patients before and after receiving dietary guidance. The symbols are the same as in Figure 1. *P<0.05.

PYY cell density: The densities of PYY cells in the controls and patients are summarized in Table 2 and illustrated in Figure 3. The density of PYY cells in the left colon of IBS patients increased significantly (*P*=0.04) after receiving dietary guidance. The densities of PYY cells also increased in the total colon and the right colon following dietary guidance, but the change did not reach the cutoff for statistical significance (*P*=0.06 and *P*=0.1, respectively). The densities of PYY cells in controls and IBS subtypes before and after receiving guidance are listed in Table 3.

#### Rectum

The densities of serotonin, PYY and somatostatin cells in controls and patients are reported in Table 4.

**Figure 4:**
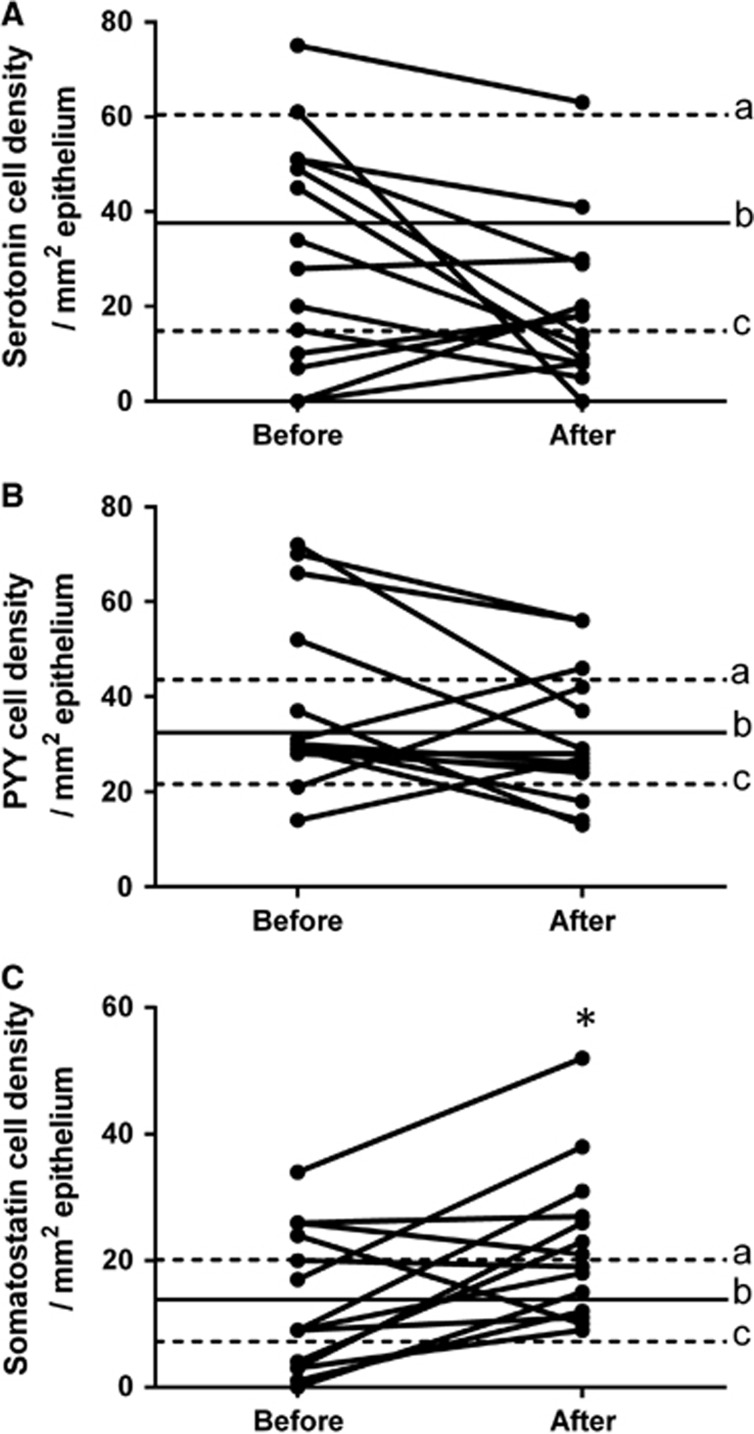
Cell densities of (**A**) serotonin, (**B**) PYY and (**C**) somatostatin in the rectum of IBS patients before and after receiving dietary guidance. The symbols are the same as in Figure 1. *P<0.05.

Serotonin cell density: The serotonin cell density in the rectum of IBS patients did not differ significantly (*P*=0.06) between before and after receiving dietary guidance (Figure 4A).

PYY cell density: The density of PYY cells in the rectum of IBS patients did not change significantly (*P*=0.13) after receiving dietary guidance (Figure 4B).

Somatostatin cell density: The somatostatin cell density of IBS patients increased significantly (*P*=0.01) after receiving dietary guidance (Figure 4C).

## Discussion

The dropout rate in the present study was 52%, somewhat higher than previously reported rates (33–48%).^35, 39, 40, 41, 42^ This higher dropout rate could be explained by the demanding design of the study, involving two colonoscopies and requiring the patients to follow a strict diet for at least 3 months. Moreover, an additional 20% of the patients were excluded from the study for other reasons (diagnosis of celiac disease, pregnancy, moving abroad, noncompliance, gastroenteritis and technical difficulties experienced during the colonoscopy). However, despite the small number of the sample who completed the study, changing the diet in these patients was shown to have a significant impact on the large intestinal endocrine cells. It is worth mentioning that neither age nor gender affects the densities of the endocrine cells in the large intestine.^36, 43^

A previous study involving the same cohort of IBS patients investigated in the present study^36^ found that the total endocrine cells, as detected by chromogranin A, changed in the colon, but not in the rectum. The present findings show that these changes in the colon are brought about by changes in serotonin and PYY cells. The elevation in the density of somatostatin cells in the rectum seen in the present study does not seem to have affected the total densities of endocrine cells.

Serotonin is known to activate the submucosal sensory branch (Meissner's plexus) of the enteric nervous system that conveys sensation from the GI tract to the central nervous system and modulates the visceral sensitivity of the GI tract.^7, 44, 45, 46, 47^ Serotonin also stimulates the motility of the large intestine, and accelerates the transit time through both the small and large intestines.^44, 45, 46, 47, 48, 49, 50, 51, 52^ The serotonin cell density has been found to be low in patients with IBS.^11^ After receiving dietary guidance in the present study, the serotonin cell density in the colon increased significantly toward the values for the control subjects. The serotonin cell density in the rectum of IBS patients has been reported to be normal.^9^ The serotonin cell density in the rectum remained unchanged in IBS patients in the present study after receiving dietary guidance.

PYY stimulates the absorption of water and electrolytes, and is considered to be a major regulator of the ‘ileal brake'.^7, 53^ The cell density of PYY has been reported to be low in the colon^11^ and rectum^9^ of IBS patients. The cell density of PYY increased significantly only in the left colon toward the values of control subjects, after receiving dietary guidance. The increase in the PYY cell densities in the total and right colon was not significant, and this could be a type II error because of the small sample size being studied. The cell densities of PYY did not significantly change in the rectum.

A previous study on the same cohort of IBS patients showed a significant improvement in pain (*P*<0.001) and diarrhea (*P*<0.05) domains but not in constipation using Birmingham IBS symptom questionnaire after receiving dietary guidance compared with before guidance.^34^ It is possible that the significant changes in the cell densities of serotonin and PYY in IBS-D patients could be related to the observed symptomatic changes after receiving dietary guidance in the mentioned article. The different changes in the cell densities in different parts of the colon could be a type II error because of the small sample size. In the same manner, the nonsignificant changes in the cell densities in IBS-C patients might be related to the nonsignificant change in the constipation domain of Birmingham IBS symptom questionnaire after receiving dietary guidance.^34^

Somatostatin inhibits intestinal motility and exocrine and neuroendocrine secretion.^8, 54^ The rectal somatostatin cell density has been found to be higher in patients with IBS.^9^ The somatostatin cell density in the rectum increased significantly after receiving dietary guidance. This increase in somatostatin cell density is difficult to explain. It is possible that it may be caused by a compensatory reaction to the increased cell density of PYY after receiving dietary guidance.

The changes in the endocrine cell densities after receiving dietary guidance differed between the colon and rectum, probably because of their different functions: the colon absorbs water, sodium and some fat-soluble vitamins, whereas the rectum acts only as a fecal reservoir before defecation.^13^

Nutrients in the lumen of the GI tract are the main triggers for the endocrine cells releasing various GI hormones that control and regulate several functions of the GI tract.^8, 55^ The stem cells in the GI tract require between 2 and 6 days to differentiate into different endocrine cells.^56, 57^ It can therefore be speculated that changing the pattern of food intake can alter the differentiation of the endocrine cells.

In conclusion, this study is the first to show that the densities of the endocrine cells in the large intestines are affected by the type of food consumed. A change in diet of IBS patients following the provision of dietary guidance can normalize the densities of these endocrine cells and recover their malfunctioning, and may have resulted in the improvement of IBS symptoms.

## Figures and Tables

**Table 1:** Demographic characteristics of participants

*IBS subtype/controls*	*Number*	*Age (mean)*^a^	*Females/males*
IBS-D	6	30–44 (37.2)	2/4
IBS-C	5	27–42 (32.4)	4/1
IBS-M	2	21–23 (22)	2/0
Controls	13	26–70 (54)	9/4

Abbreviations: IBS, irritable bowel syndrome; IBS-C, IBS patients with constipation as a predominant symptom; IBS-D, IBS patients with diarrhea as the predominant symptom; IBS-M, IBS patients with mixed diarrhea and constipation.

a Years.

**Table 2:** Densities of serotonin- and PYY-immunoreactive cells in the total colon, right colon and left colon of control subjects and of IBS patients before and after receiving dietary guidance

*Hormone/location*	*Cell density (cells/mm*^*2*^)			
	*Control*	*Before guidance*	*After guidance*	P-*value*
*Serotonin*				
Total colon	46.8±8.9	10.5±2.1	22.6±3.2	0.007^b^
Right colon	25.9±5.4	1.2±0.8	10.7±1.6	<0.0001^c^
Left colon	20.9±4.8	8.9±1.7	10.6±1.9	0.53
*PYY*				
Total colon	11.6± 1.8	10.8±1.7	16.8±2.1	0.06
Right colon	3.9±0.8	2.9±0.8	5.5±1.1	0.1
Left colon	7.7±1.4	7.9±1.0	11.5±1.1	0.04^a^

Abbreviations: IBS, irritable bowel syndrome; PYY, peptide YY.

Data are presented as mean±s.e.m.

a *P*<0.05, ^b^*P*<0.01 and ^c^*P*<0.0001.

**Table 3:** Densities of serotonin- and PYY-immunoreactive cells in the total colon, right colon and left colon of controls and patients with different IBS subtypes before and after receiving dietary guidance

*Hormone/location*	*Endocrine cell densities (cell/mm*^*2*^)		*Endocrine cell densities (cell/mm*^*2*^)		P-*value, before guidance* vs *controls*	P-*value, after guidance* vs *controls*	P-*value, before* vs *after guidance*
	*Control*	*IBS subtype*	*Before guidance*	*After guidance*			
*Serotonin*							
Total colon	46.8±8.9	IBS-D	11.17±3.4	19.83±3.7	0.009^b^	0.2	0.12
		IBS-C	11.75±3.8	22.75±8.1	0.06	0.26	0.24
		IBS-M	6.0±2.0	30.5±0.5	0.16	0.66	0.06
Right colon	25.9±5.4	IBS-D	2.16±1.6	10.3±1.7	0.002^b^	0.22	0.009^b^
		IBS-C	0.75±0.48	11.5±4.7	0.03^a^	0.43	0.1
		IBS-M	0	10.7±2.6	0.02^a^	0.42	0.05
Left colon	20.9±4.8	IBS-D	9.0±2.9	9.5±2.1	0.39	0.59	0.89
		IBS-C	10.0±3.1	9.0±3.7	0.42	0.33	0.83
		IBS-M	6.0±2.0	18.0±3.0	0.39	0.9	0.25

*PYY*							
Total colon	11.6±1.8	IBS-D	11.17±2.4	19.17±2.5	0.9	0.051	0.04^a^
		IBS-C	12.5±3.5	11.5±3.3	0.9	0.9	0.83
		IBS-M	6.5±3.5	20.5±7.5	0.43	0.37	0.42
Right colon	3.9±0.8	IBS-D	3.83±1.3	6.0±1.4	0.9	0.31	0.39
		IBS-C	3.5±1.7	4.3±2.5	0.9	0.9	0.78
		IBS-M	0.33±0.33	6.3±2.4	0.09	0.8	0.15
Left colon	7.7±1.4	IBS-D	7.3±1.7	13.2±1.4	0.9	0.04^a^	0.002^b^
		IBS-C	9.4±1.5	8.6±1.6	0.95	0.9	0.72
		IBS-M	6.0±3.0	13.5±3.5	0.87	0.25	0.45

Abbreviations: IBS, irritable bowel syndrome; IBS-C, IBS patients with constipation as a predominant symptom; IBS-D, IBS patients with diarrhea as the predominant symptom; IBS-M, IBS patients with mixed diarrhea and constipation; PYY, peptide YY.

Data are presented as mean±s.e.m.

a *P*<0.05 and ^b^*P*<0.01.

**Table 4:** Densities of serotonin-, PYY- and somatostatin-immunoreactive cells in the rectum of control subjects and of IBS patients before and after receiving dietary guidance

*Hormone*	*Endocrine cell density (cell/mm*^*2*^)			P-*value, before* vs *after guidance*
	*Control*	*Before guidance*	*After guidance*	
Serotonin	37.7±10.5	31.9±6.4	19.6±4.5	0.06
PYY	32.5±5.1	38.4±5.0	31.5±3.7	0.13
Somatostatin	13.5±3.0	13.2±3.0	22.3±3.2	0.01^a^

Abbreviations: IBS, irritable bowel syndrome; PYY, peptide YY.

Data are presented as the mean±s.e.m.

a *P*<0.05.
